# Supplementary material for: Improving object detection quality with structural constraints
Source: PLoS One. 2022 May 18;17(5):e0267863. doi: 10.1371/journal.pone.0267863 (PMC9116628; doi:10.1371/journal.pone.0267863)
Supplement: Attachment — Submitted filename: r2_1-reply.pdf [file pone.0267863.s001.pdf]

Improving object detection quality with structural constraints

Zihao Rong[1], Shaofan Wang[1], Dehui Kong[1], Baocai Yin[1]

[1] Beijing Key Laboratory of Multimedia and Intelligent Software Technology,
Faculty of Information Technology, Beijing University of Technology, Beijing 100124, China

Response to Reviewer

Comment 1: "In this paper, the two additional loss functions are directly added to the original loss function, such as formula 4, but in the following 3.3, it is explained that they are used in different branches, and they are inconsistent."

Reply: Thanks for this comment. Since that we confine the scope to modern object detection networks which allow end-to-end training, it's possible to calculate different loss terms for different network outputs and then sum these terms together to calculate back-propagation. Thus, it's possible to calculate Fisher loss and equi-proportion loss on different network branches, and add them onto the original loss to guide network training. Therefore, Equition (4) and Section 3.3 are consistent. As for earlier object detectors, this indeed may not hold, like DPM and R-CNN, where components of the detectors are trained separately.

Comment 2: "After adding the loss function, how to train the network? Is it to train with the classification loss first, and then use the Fisher loss for fine-tuning? It needs to be explained in detail here."

Reply: Thanks for this comment. We use the original loss and our structural constraint losses to jointly guide network training, which means the sum of the original loss and structural constraint losses is used directly to calculate back-propagations. We think this could lead the network parameters closer to global optimum. We clarified this at the end of Section 3.2. Your suggestion of training with individual loss iteratively is very interesting, and we hope we could try it in our future works.

Comment 3: "The super parameters in the experimental settings, such as the setting of IOU threshold, directly give specific values, but do not explain how to select this parameter value. Further clarification is required."

Reply: Thanks for this suggestion. All hyper-parameters, including the IoUs, are set to default values of MMDetection configuration files. These values proved to be proper and led to acceptable network performances during MMDetection benchmarking. We inserted a clarification of this at the beginning of Section 4.1.

Comment 4: "What is the difference between the Fisher loss added in this paper and the Fisher discriminative layer in reference paper 2."

Reply: Thanks for this comment. Reference paper 2's RIFD-CNN and our Fisher loss express Fisher discrimination in different ways: RIFD-CNN constrains the relations between individual training samples and class-wise means, and the relations between class-wise means and the global mean; Fisher loss constrains purely pairwise training sample relations. Besides, RIFD-CNN does not constrain inter-class relations, while Fisher loss constrains both inter- and intra-class relations. We clarified this at the end of Section 2.1.
